# Janus adhesive bio-patches with targeted drug delivery enabled anti-bacteria and pro-angiogenesis for dura mater repair

**DOI:** 10.1016/j.mtbio.2025.101484

**Published:** 2025-01-11

**Authors:** Yirizhati Aili, Pengfei Wei, Xueqiao Yu, Guofeng Fan, Nuerailijiang Maimaitiaili, Yunhuan Li, Siqi Liu, Yiqian Huang, Bo Zhao, Zengliang Wang, Hu Qin, Yongxin Wang

**Affiliations:** a Department of Neurosurgery, The First Affiliated Hospital of Xinjiang Medical University, No.393 Xinyi Road, Urumqi, Xinjiang, 830054, China; b Key Laboratory of Precision Diagnosis and Clinical Transformation of Nervous System Tumors, Xinjiang Medical University, No.393 Xinyi Road, Urumqi, Xinjiang, 830054, China; c Beijing Biosis Healing Biological Technology Co., Ltd, No.29 Yongda Road, Beijing, 102600, China

**Keywords:** Dural injury, Cerebrospinal fluid leakage, SIS, Bio-adhesive, Tissue sealing

## Abstract

Dural injuries often result in severe complications such as cerebrospinal fluid (CSF) leakage, intracranial infections, and brain herniation, which significantly impact patient recovery and quality of life. Conventional dural repair materials, which rely on suturing to peripheral tissues, fail to promote tissue regeneration and provide sufficient CSF leakage prevention, leading to suboptimal outcomes. To address these limitations, we developed a Janus adhesive bio-patch with both antibacterial and pro-angiogenic properties to enhance dura mater repair. This bio-patch consisted of a polyacrylic acid (PAA) adhesive gel layer loaded with vancomycin and magnesium carbonate (MgCO_3_), integrated onto a small intestinal submucosa (SIS) extracellular matrix. It exhibited a burst strength of 20.50±2.89kPa, effectively sealing CSF leaks, while demonstrating excellent antibacterial efficacy (∼99%) and significant enhanced angiogenesis (3.47-fold higher than the control). In rat, rabbit, and dog dural injury models, the bio-patch adhered seamlessly to the injury site, successfully preventing leaks and promoting tissue regeneration. These results highlighted the Janus adhesive bio-patch as a promising solution for improving dural repair in neurosurgery, offering a safer and more effective alternative to conventional suturing techniques.

## Graphical abstract

**Figure:**
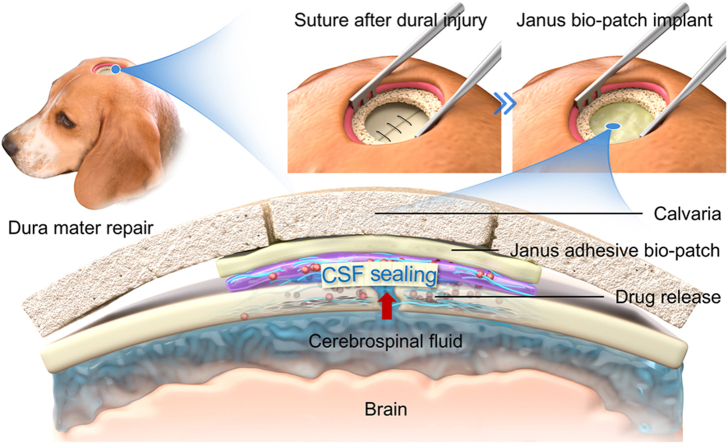

## 1 Introduction

Dura mater is a resilient membrane that encases the brain and spinal cord, playing a crucial role in protecting the central nervous system [1]. Dural tears occur in approximately 20% of craniotomy, significantly increasing the risk of both localized and systemic postoperative complications. In the United States, the average cost of surgically repairing dural tears is $18,499 per patient annually, with this figure steadily increasing [2, 3]. Dural injuries can result from trauma, tumor resection, cerebrovascular interventions, and craniotomy procedures [4, 5], such injuries not only predispose patients to cerebrospinal fluid (CSF) leakage, but also elevate the risk of severe complications, including intracranial infections and brain herniation, underscoring the clinical importance of developing improved repair materials.

Conventional repair techniques, including autologous tissue transplantation, allogeneic grafts, and synthetic materials, have been widely used but face notable limitations. Autologous grafts, while immune-compatible, require additional surgery, risking donor site morbidity [6, 7]. Allogenic grafts, though avoiding secondary trauma, carry risks of immune rejection and disease transmission, limiting their clinical use. Synthetic materials such as polyvinyl alcohol and polytetrafluoroethylene, provide good mechanical strength but often suffer from poor biocompatibility, lack antibacterial properties, and fail to promote tissue regeneration, leading to chronic inflammation and suboptimal outcomes [[8], [9], [10], [11]].

Recently, porcine small intestinal submucosa (SIS), a naturally derived extracellular matrix, has gained attention in dural repair due to its excellent biocompatibility, biodegradability, and ability to serve as a scaffold for cell proliferation and tissue regeneration [[12], [13], [14]]. However, SIS lacks intrinsic antibacterial and adhesive properties [15, 16], limiting its ability to prevent postoperative infections and CSF leakage. Therefore, enhancing SIS with antibacterial and pro-angiogenic functions has become a key focus in biomaterial research for dural repair.

In this study, we engineered a Janus adhesive bio-patch using SIS as the substrate, functionalized with polyacrylic acid (PAA) hydrogel, vancomycin (Van), and magnesium carbonate (MgCO_3_). PAA hydrogel was selected for its strong adhesion and biocompatibility, enabling effective CSF leakage prevention [17, 18]. Vancomycin (Van), a potent antimicrobial agent, was incorporated to inhibit bacterial growth and reduce infection risk [19, 20]. Magnesium ions (Mg^2+^), known for their role in tissue regeneration, promote angiogenesis, cell proliferation, and anti-inflammatory responses, further enhancing tissue repair outcomes [[21], [22], [23], [24], [25]].

**Scheme 1:**
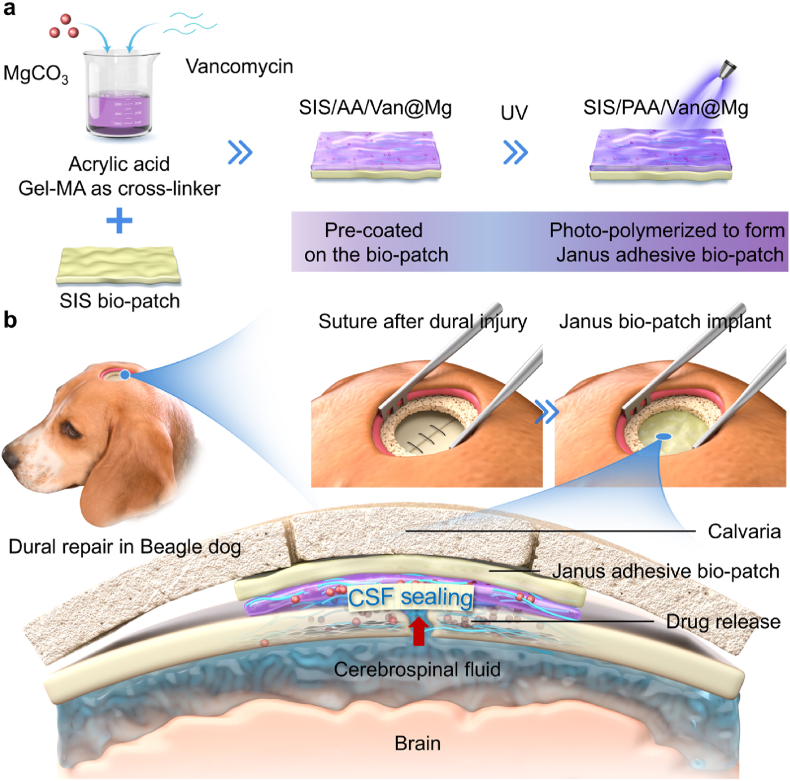
(a) Acrylic acid pre-polymerized solutions were combined with bioactive components MgCO_3_ and vancomycin, and then coated onto the SIS bio-patch. After ultraviolet light crosslinking, the Janus adhesive bio-patch was fabricated. (b) Upon application to dural injury, the Janus adhesive bio-patch was capable of sealing CSF leakage while releasing vancomycin and Mg^2+^ to facilitate tissue regeneration.

Our results demonstrated that the SIS/PAA/Van@Mg bio-patch exhibited robust antibacterial efficacy, strong tissue adhesiveness, and superior angiogenesis in vitro. In vivo studies using rat, rabbit, and beagle dog models revealed effective CSF sealing, enhanced tissue regeneration, and reduced postoperative complications (Scheme 1). This multifunctional bio-patch provides a promising solution for improving clinical outcomes in dural repair, offering a safer and more efficient alternative to conventional methods.

## 2 Materials and methods

### 2.1 Materials

Acrylic acid, vancomycin, MgCO_3_ were obtained from Aladdin (Shanghai, China). α-Ketoglutaric acid was purchased from Sigma-Aldrich (USA). Methacrylated gelatin (Gel-MA) was purchased from Sigma, SIS was provided by Beijing Biosis Healing Biological Tech. Co., Ltd (Beijing, China). Other chemical reagents except otherwise statement were of chemical grade, and supplied by Beijing Chemical Reagent Co., Ltd. (China).

### 2.2 Fabrication of the Janus adhesive bio-patch

Acrylic acid (30.0wt.%), Gel-MA (1.0wt.%), α-ketoglutaric acid (0.2wt.%) was dissolved in deionized water, and coated onto the SIS at a rate of 50μL/cm^2^. The SIS/PAA bio-patch was then prepared by ultraviolet (UV) irradiation (300W) for 30 minutes at the wavelength of 365nm. Similarly, the SIS/PAA/Van, SIS/PAA@Mg, and SIS/PAA/Van@Mg was fabricated by incorporating vancomycin (0.1wt.%), MgCO_3_ (1.0wt.%) into the pre-polymerized solutions, followed by ultraviolet radiation for 30 minutes to produce the Janus adhesive bio-patches.

### 2.3 Characterization of the Janus adhesive bio-patch

Cross-sectional morphological images were obtained by fracturing the bio-patch in liquid nitrogen and examining the fracture surface using scanning electron microscope (SEM, S4800, Hitachi, Japan) after platinum sputter-coating. Elemental mappings were performed using energy-dispersive spectroscopy (EDS). Fourier-transform infrared (FT-IR) spectra were recorded with an ATR-FT-IR spectrometer (Thermo Nicolet iS5, Thermo Fisher Scientific, USA).

### 2.4 Drug release from the Janus adhesive bio-patch

A regular shape of the SIS/PAA/Van@Mg bio-patch (2cm×2cm) was placed in a centrifuge tube with 5mL PBS immersion, then the tube was placed at a shaking water bath at a rate of 50rpm and 37°C. At one-, four-, and seven-day, the supernatants were retrieved and sent to measure the absorbance at 280nm using a UV–Vis spectrophotometer. The vancomycin concentration was calculated based on a vancomycin standard curve.

Similarly, Mg^2+^ release was conducted as follows, except that the retrieving time was one, four-seven, 14-, and 21-day. The collected supernatants were sent to inductively coupled plasma optical emission spectrometer (ICP-OES, Shimadzu ICPE-9800, Japan) to detect the Mg^2+^ concentrations.

### 2.5 Tissue adhesion for the Janus adhesive bio-patch

Burst strength test was according to ASTM F2392. A 5mm defect was created in a 3cm segment of porcine colon using a biopsy punch. The bio-patches (2cm×2cm) were placed over the defect and allowed to adhere for 60 minutes at room temperature. A pressure pump applied pressure at a rate of 2 mL min^−1^ until leakage happened, at which point the burst strength was recorded.

Shear strength test was according to ASTM F2255. Porcine skins were cut into 4cm×2.5cm pieces, then the bio-patches were applied to one of the porcine skins, covering an area of 2cm×2cm. The two pieces were then bonded in opposing directions at room temperature. After 60 minutes, a universal testing machine was used to measure the force-displacement curve in the shear direction at a rate of 5 mm min^−1^. Shear strength (Pa) was calculated as:

$$\text{Shear}\;\text{strength}\;\left(\text{Pa}\right)=\frac{F_{\max}}{\text{Area}}\times 100\%$$

Interfacial toughness test was according to ASTM F2256. Porcine skins were cut into 4cm×2.5cm pieces, then the bio-patches were applied to one of the porcine skins, covering an area of 2.5cm×1cm. The two pieces were then aligned and adhered at room temperature. After 60 minutes, a universal testing machine was used to measure the force-displacement curves in the shear direction at a rate of 5 mm min^−1^. Interfacial toughness (J m^−2^) was calculated as:

$$\text{Interfacial}\;\text{toughness}\;\left(J\;m^{-2}\right)=\frac{{2\times F}_{\max}}{W\text{idth}}\times 100\%$$

### 2.6 Cyto-compatible studies of the Janus adhesive bio-patch

#### 2.6.1 Hemolysis assay

According to ISO10993, a 2cm×3cm bio-patch was placed in a 2mL centrifuge tube with 1mL of PBS and incubated at 37°C for 30 minutes. After incubation, 900μL of the extract was mixed with 200μL of whole rabbit blood. For controls, 900μL of PBS mixed with 200μL of whole rabbit blood was used as the negative control, and 900μL of Triton X-100 mixed with 200μL of whole rabbit blood was used as the positive control. All samples were incubated together at 37°C for one hour. The mixtures were then centrifuged at 4000rpm for one minute using a benchtop high-speed centrifuge. Photographs were taken, and 100μL of the supernatant was transferred to a 96-well plate for absorbance measurement at 540nm using a microplate reader. Hemolysis rate (%) was calculated as:

$$\text{Hemolysis}\;\text{ratio}\;\left(\%\right)=\frac{A_{M}‐A_{N}}{A_{P}‐A_{N}}\times 100\%$$

The A_M_, A_N_, and A_P_ represented the absorbance values of the material groups, negative control, and positive control, respectively.

#### 2.6.2 Compatibility with L929 fibroblasts

In accordance with ISO10993 biological testing standards, the SIS-based bio-patches were immersed in cell culture medium at a ratio of 6 cm^2^ mL^−1^. After 24 hours, the extract was used to co-culture L929 fibroblasts (1000 cells per well) in a 48-well plate. The co-culture was maintained for 24 hours. Cell viability was assessed using the CCK-8 reagent, with absorbance measured at 450nm using a microplate reader (Bio-Rad 680, USA). Additionally, live/dead cell imaging was performed using a confocal laser scanning microscope (CLSM, TCS SP8, Leica, Germany) after staining with calcein-AM/PI fluorescent dye (Aladdin, China).

### 2.7 Antibacterial assays of the Janus adhesive bio-patch

#### 2.7.1 Morphological observation

A 2cm×3cm bio-patch was placed in a 60mm diameter Petri dish containing 50μL of bacterial suspension (1×10⁵CFU/mL) and 10mL of TSB medium. The dish was incubated at 37°C for 24 hours. Following incubation, the medium was aspirated, and the bio-patches were rinsed three times with deionized water. The bio-patches were then fixed in 2.5% glutaraldehyde solution for 12 hours. After fixation, the glutaraldehyde solution was discarded, the bio-patches were rinsed twice with 75% ethanol and allowed to dry completely before sending to SEM.

#### 2.7.2 Inhibition zone

The bio-patch was cut into circular disks, each with a diameter of 1cm. A 100μL aliquot of *S.aureus* suspension (1×10⁵CFU/mL) was applied onto a TSA agar plate, and spread evenly using a sterile spreader. Each bio-patch disk was then placed in a specific area on the agar plate. The plate was incubated at 37°C in a temperature and humidity-controlled incubator for 24 hours. After incubation, photographs of the plates were taken for analysis.

#### 2.7.3 Bacterial viability

A 2cm×2cm sample was placed into a petri dish (60mm in diameter) containing 50μL of a bacterial suspension at a concentration of 1×10⁵CFU/mL and 10mL of TSB culture medium. The dish was then incubated at 37°C for 24 hours. After incubation, 100μL of the solution was transferred to a 96-well plate, and the absorbance was measured at 600nm. Additionally, 1mL of the solution was transferred to a 1.5 mL EP tube, and photographs were taken using a camera. Antibacterial rate (%) was calculated as:

$$\text{Antibacterial}\;\text{rate}\;\left(\%\right)=\frac{A_{C}‐A_{M}}{A_{C}}\times 100\%$$

The A_C_ and A_M_ represented the absorbance values of the control groups, and bio-patch control, respectively.

#### 2.7.4 Biofilm formation

A 2cm×2cm sample was placed into a petri dish (60mm in diameter) containing 50μL of a bacterial suspension at a concentration of 1×10⁵CFU/mL and 10mL of TSB culture medium. The dish was then incubated at 37°C on a shaker at 50rpm for 24 hours. After incubation, the sample was removed and placed into a 24-well plate, with each well receiving 120μL of 0.1% crystal violet solution (dissolved in methanol) to stain the biofilm. The plate was left at room temperature for 10 minutes before the crystal violet solution was removed. The wells were washed three times with PBS and air-dried. Subsequently, 200μL of 95% ethanol was added to each well to elute the crystal violet dye from the biofilm. The plate was left at room temperature for 15 minutes, and images were captured using a camera. Finally, 100μL of the eluted solution was transferred to a 96-well plate, and the absorbance at 540nm was measured to assess biofilm formation. Biofiom biomass (%) was calculated as:

$$\text{Biofiom}\;\text{bioss}\;\left(\%\right)=100‐\frac{A_{C}‐A_{M}}{A_{C}}\times 100\%$$

The A_C_ and A_M_ represented the absorbance values of the control groups, and bio-patch control, respectively.

### 2.8 Angiogenic assays of the Janus adhesive bio-patch

#### 2.8.1 Immunofluorescent staining of VEGF

To evaluate the angiogenic potential of the extract, human umbilical vein endothelial cells (HUVECs) were utilized. A density of 5,000 HUVECs were seeded in a 48-well plate and incubated with the extract for seven days, with the medium refreshed every other day. After incubation, the cells were stained with a primary VEGF antibody (ab32152, Abcam, UK), FITC-labelled F-actin and a fluorescent secondary antibody. After a 30-min incubation in the dark, VEGF expression within the cells was observed under CLSM. Fluorescence intensity was semi-quantitatively analyzed using Image Pro Plus software.

#### 2.8.2 Tube formation

Matrigel (Corning, USA) was used to create a substrate for cell seeding in cell culture plates. A density of 5,000 HUVECs were seeded onto the Matrigel and incubated with the extract for 12 hours. Tubular network formation was then observed under an optical microscope. The spacing between the tubular networks was semi-quantitatively measured using Image J software.

#### 2.8.3 Cell scratch assay

A density of 5,000 HUVECs was seeded into 24-well plates. Once the cells reached approximately 80% confluence, a sagittal scratch of 200μm in width was created using a pipette tip. The cells were then cultured with the bio-patch's extract. After 24 hours, cell migration was observed under a microscope. The trace distance of cell migration was analyzed semi-quantitatively using ImageJ software.

### 2.9 Dural mater repair studies in vivo

All the animal experiments were complied with the guidelines of the Tianjin Medical Experimental Animal Care, and animal protocols were approved by the Institutional Animal Care and Use Committee of Yi Shengyuan Gene Technology (Tianjin) Co., Ltd (protocol number YSY-DWLL-2024433, 2024434, 2024435).

#### 2.9.1 Dural repair in the infected injury on Sprague Dawley rats

After anesthetizing the rats, a 5mm circular defect was created in the skull using a trephine drill, and the excised bone fragment was removed. The dura mater at the base of the skull was then incised in a straight line using a scalpel. The rats were randomly divided into four groups: 1) Control group, where *S.aureus* (∼10^5^CFU) was introduced into the wound without further treatment. 2) *S.aureus* (∼10^5^CFU) followed by implantation of the SIS bio-patch. 3) *S.aureus* (∼10^5^CFU) followed by implantation of the SIS/PAA/Van bio-patch. 4) *S.aureus* (∼10^5^CFU) followed by implantation of the SIS/PAA/Van@Mg bio-patch. The wounds were then sutured hierarchically without suturing the dural injury to study the antibacterial efficacy. At one- and four-week post-surgery, the rats were euthanized, and tissues from the dural injury site were collected for histological and immunofluorescence analysis, including H&E, TNF-α (ab220210, Abcam, UK), CD163 (ab182422, Abcam, UK), and DAPI staining. Additionally, one week after surgery, a 1mm^3^ granulation tissue was collected, placed in 1mL sterile saline for two hours, and 50μL of the supernatant was spread onto a sterile agar plate. The plate was incubated at 37°C for 24 hours to assess bacterial colony formation. Giemsa staining was performed on the dura mater tissues collected at one-week to identify any residual exogenous bacteria. Fluorescence intensity was semi-quantitatively analyzed using Image Pro Plus software.

#### 2.9.2 Dural repair in the injury on New Zealand rabbits

Following anesthesia, New Zealand rabbits underwent a 10mm circular skull defect procedure using a trephine drill, with the excised bone fragment removed. The dura mater beneath the calvaria was then incised along a straight line using a scalpel. The rabbits were then randomly divided into three groups: 1) Control group with suturing only. 2) Suturing followed by SIS implantation. 3) Suturing followed by SIS/PAA/Van@Mg implantation. The wounds were then sutured in layers, and this model was focused on studying the tissue regeneration outcomes. At two- and four-week post-surgery, the rabbits were euthanized, and tissues from the dura mater injury site were collected. These tissues were fixed in 4% paraformaldehyde for 24 hours and then sectioned for histological and immunofluorescence analysis, including H&E, Masson's trichrome, TNF-α (7124-MSM1-P1BX, ThermoFisher, USA), TGF-β (MA1-21595, ThermoFisher, USA), and VEGF (MA5-13182, ThermoFisher, USA). Collagen formation and fluorescence intensity were assessed semi-quantitatively using Image Pro Plus software. At four-week post-surgery, blood samples were collected for hematological analysis. Moreover, heart, liver, spleen, lungs, and kidney tissues were examined through pathological staining.

#### 2.9.3 Pre-clinical dural repair in the injury on beagle dogs

Under anesthesia, the Beagle dogs underwent a craniotomy to create a 20mm circular cranial defect using a trephine drill, with the excised bone fragment discarded. The dura mater under the calvaria was incised along a straight line using a scalpel. The dogs were randomly divided into three groups: 1) Control, which received suture only. 2) Suture followed by SIS implantation. 3) Suture followed by SIS/PAA/Van@Mg implantation. The wounds were then sutured hierarchically. Electrocardiogram (ECG) monitoring was performed for the SIS/PAA/Van@Mg group before surgery, at four- and eight-week post-surgery. At eight-week post-surgery, the dogs were euthanized, and tissues from the dura mater injury site were collected for histological and immunofluorescence analysis, including Masson's trichrome, VEGF (MA1-16629, ThermoFisher, USA), collagen (ab24137, Abcam, UK), DAPI, and TLR2 (ab9100, Abcam, UK), CD4 (MA5-28357, ThermoFisher, USA), and DAPI. Collagen expression (based on collagen fluorescence staining) and vascularization level (based on VEGF fluorescence staining) were analyzed semi-quantitatively using analysis Image Pro Plus software, based on collagen and VEGF fluorescence staining, respectively. At eight-week post-surgery, heart, liver, spleen, lung, and kidney tissues were also collected for pathological staining.

### 2.10 Statistical analysis

All quantitative data was represented as mean±standard deviation (SD). Statistical analysis was carried out using Graphpad9.4 software by one-way analysis of variance (ANOVA). Differences between groups of ^∗^*P*<0.05 were considered statistically significant, ^∗∗^*P*<0.01 were considered highly significant, ^∗∗∗^*P*<0.001 were considered very highly significant.

## 3 Results and discussion

### 3.1 Preparation and characterization of the Janus adhesive bio-patch

**Fig. 1:**
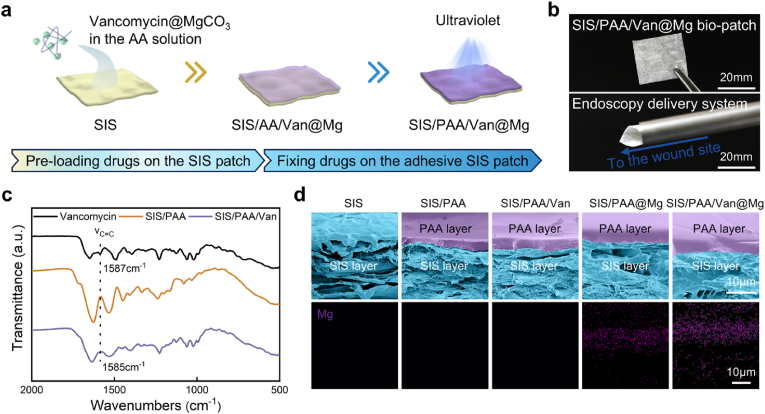
Preparation and characterization of the Janus adhesive bio-patch. (a) Schematic illustration depicting the preparation method of the SIS/PAA/Van@Mg bio-patch. The process started with the pre-loading of vancomycin and MgCO_3_ particles into an acrylic acid solution, followed by slurry coating and subsequent photo-polymerization on SIS substrates. (b) Gross appearance of the SIS/PAA/Van@Mg bio-patch and its potential applications in endoscopic procedures. (c) FT-IR spectra of the SIS/PAA/Van@Mg bio-patch. (d) SEM and elemental mapping of Mg of the SIS/PAA/Van@Mg bio-patch.

We developed a Janus adhesive dural repair bio-patch using α-ketoglutaric acid, a metabolite from the tricarboxylic acid cycle, as a photo-initiator [26]. Acrylic acid, vancomycin, and MgCO_3_ were incorporated into a pre-polymerized solution with Gel-MA serving as the crosslinker. The mixture was coated onto the SIS substrate and subjected to UV irradiation (300W) for 30 minutes, inducing free radical polymerization and crosslinking to form the SIS/PAA/Van@Mg bio-patch (Fig. 1a). The prepared SIS/PAA/Van@Mg bio-patch exhibited excellent flexibility, enabling its compatibility with various delivery devices and enhancing its applicability in minimally invasive endoscopic procedures (Fig. 1b). FT-IR spectra revealed the successful integration of vancomycin into the PAA gel coating, as evidenced by a characteristic peak at 1585cm^−1^, corresponding to the C=C stretching vibration of the vancomycin aromatic ring (Fig. 1c) [27]. SEM images further showed that a uniform PAA coating on the SIS substrate, with elemental mapping validating the homogenous distribution of Mg within the bio-patch (Fig. 1d). Drug release behavior was assessed by immersing the bio-patch in PBS. Both vancomycin and Mg^2+^ were released in a sustained manner over seven days, with initial concentrations of 30.57±5.72ppm for vancomycin and 228.22±50.08ppm for Mg^2+^ on the first day (Fig. S1). These concentrations were well above the reported thresholds required for antibacterial (8ppm) and angiogenic (120ppm) activities [28, 29], highlighting the therapeutic efficacy of the SIS/PAA/Van@Mg bio-patch. Collectively, these results demonstrated that the SIS/PAA/Van@Mg bio-patch combined controlled drug release, and bioactive functionality, making it a promising candidate for dural repair applications.

### 3.2 Tissue adhesion of the Janus adhesive bio-patch

**Fig. 2:**
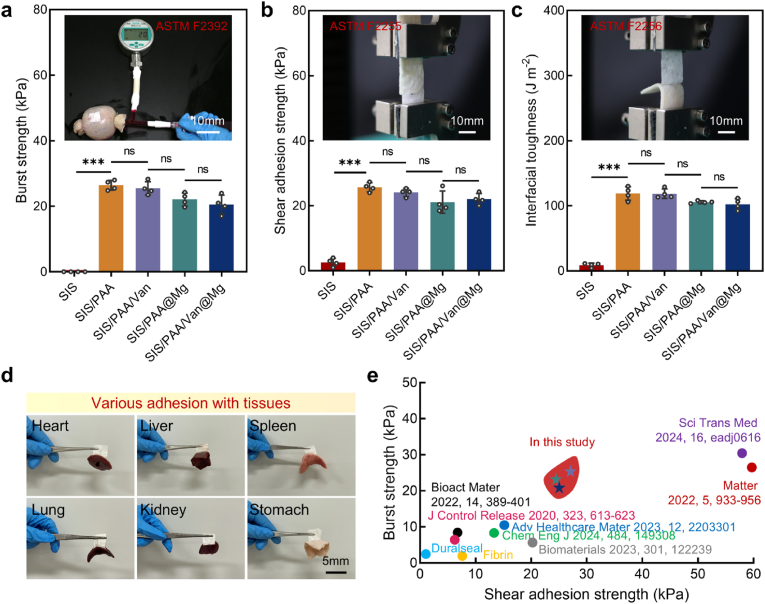
Tissue adhesion study for the Janus adhesive bio-patch. (a) Burst strength, (b) shear adhesion strength, and (c) interfacial toughness results, based on ASTM F2392, ASTMF2255, and ASTMF2256. (d) Various adhesion with tissues using the SIS/PAA/Van@Mg bio-patches. (e) Comparison with the state-of-art bio-adhesives applied in dural repair, preventing potential CSF leakage. Statistical significance and *P* values were determined by ANOVA comparison test. The data were presented as mean ± SD (n=4). ^∗∗∗^*P*<0.001.

Next, we evaluated the tissue adhesion properties of the SIS/PAA/Van@Mg dural repair bio-patch following ASTM standards for tissue adhesives (ASTM F2392, ASTM F2255, and ASTM F2256). The native SIS material exhibited poor adhesive performance, with no measurable burst strength, a shear adhesion strength of 2.58±1.26kPa, and an interfacial toughness of 8.85±3.13J m^−2^, which were insufficient for sealing CSF leakage, given that intracranial pressure can reach up to 1.96kPa [30]. Incorporation of the PAA adhesive coating significantly enhanced the adhesion performance of SIS-based materials [31]. For example, the SIS/PAA bio-patch demonstrated a burst strength of 26.43±1.52kPa, a shear adhesion strength of 25.70±1.41kPa, and an interfacial toughness of 118.90±10.40J m^−2^. Notably, the addition of vancomycin and MgCO_3_ did not compromise these adhesive properties, as the SIS/PAA/Van@Mg bio-patch maintained a burst strength of 20.50±2.89kPa, sufficient to prevent CSF leakage (Fig. 2(a-c)). In this case, when the SIS/PAA/Van@Mg bio-patch was applied to the dural injury site, it enabled robust adhesion, and then sustained released therapeutic components such as vancomycin and Mg^2+^ to achieve targeted drug delivery [32]. Moreover, the SIS/PAA/Van@Mg bio-patch exhibited versatile tissue adhesion, demonstrating strong adhesive properties across various tissues, including heart, liver, spleen, and lungs (Fig. 2d). Compared to recent advancements in neurosurgical adhesives for dural and spinal dura sealing, our Janus adhesive bio-patch exhibited superior burst and tissue adhesion strengths, positioning it as a promising candidate for clinical applications in preventing CSF leakage (Fig. 2e).

### 3.3 Cyto-compatibility of the Janus adhesive bio-patch

To evaluate the cytocompatibility of the bio-patch, hemolysis and cell viability tests were performed. Hemolysis tests were conducted by co-culturing the bio-patch extracts with red blood cells, revealing hemolysis rates below 5% for all SIS-based bio-patches, which complied with biomedical safety standards [33, 34]. In CCK-8 assays and live/dead staining of L929 fibroblasts, cell viability showed no significant differences compared to the control group, nor among the various SIS-based materials. Fluorescent staining further demonstrated well-spread, spindle-shaped fibroblasts (Fig. S2), confirming the excellent cytocompatibility of the bio-patch.

### 3.4 Antibacterial performance of the Janus adhesive bio-patch

**Fig. 3:**
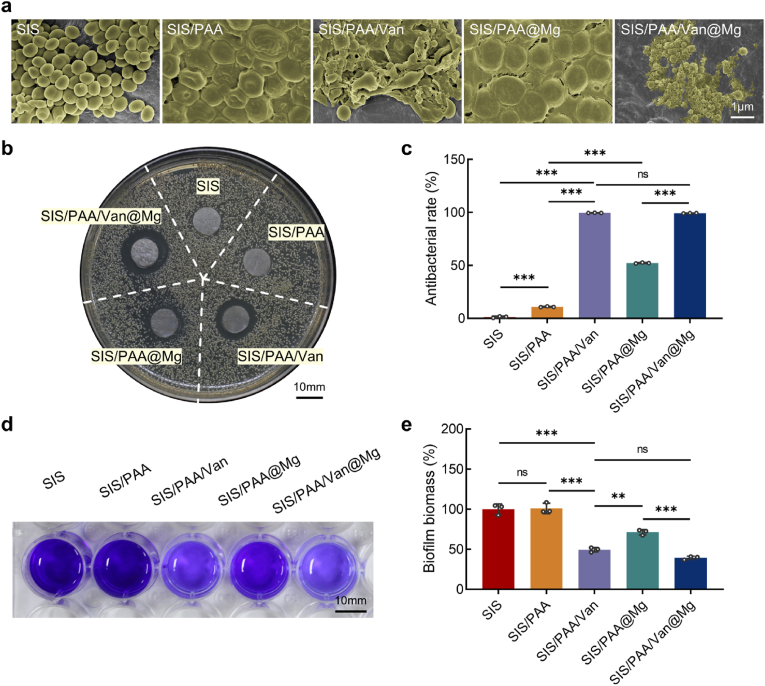
Antibacterial study for the Janus adhesive bio-patch. (a) *S.aureus* morphology after 24 hours of incubation on the various bio-patches. (b) Inhibition zones, and (c) quantitative analysis for the antibacterial rate. (d) Crystal violet staining for the biofilms with the bio-patch. (e) Biofilm biomass efficiency. Statistical significance and *P* values were determined by ANOVA comparison test. The data were presented as mean ± SD (n=3). ^∗∗^*P*<0.01, ^∗∗∗^*P*<0.001. (For interpretation of the references to colour in this figure legend, the reader is referred to the Web version of this article.)

To evaluate the antibacterial properties of the developed bio-patch, *S.aureus* was selected due to its frequent association with intracranial infections and vancomycin's proven efficacy against Gram-positive bacteria [35]. Antibacterial experiments were conducted using co-culture assays, OD_600_ measurements, inhibition zone assays, and crystal violet staining for biofilm evaluation. SEM analysis showed negligible antibacterial activity for the SIS bio-patch, with abundant *S.aureus* adhering to its surface. In contrast, the SIS/PAA bio-patch demonstrated slight bacterial deformation, likely caused by electrostatic interactions between the anionic carboxyl groups in the PAA coating and the bacterial biofilm [36]. More pronounced bacterial deformation was observed in the SIS/PAA/Van, SIS/PAA@Mg, and SIS/PAA/Van@Mg groups, attributed to vancomycin's inhibition of biofilm formation and bacterial RNA synthesis, as well as the bactericidal effects of Mg^2+^ via osmotic lysis [37, 38]. Among these groups, the SIS/PAA/Van@Mg exhibited the most significant antibacterial activity, evidenced by extensive bacterial damage (Fig. 3a). In the inhibition zone assay, the SIS/PAA/Van@Mg displayed the largest inhibition zone (Fig. 3b). Measurements of OD_600_ values further confirmed its efficacy, with bacterial inhibition rates of 1.28±0.80% for SIS, 10.90±0.40% for SIS/PAA, 99.49±0.11% for SIS/PAA@Mg, 52.18±0.48% for SIS/PAA/Van, and 99.17±0.11% for SIS/PAA/Van@Mg (Fig. 3c). Additionally, biofilm inhibition was quantified using the crystal violet assay, showing biomass reduction to 49.44±2.87% for SIS/PAA/Van, and 39.31±2.18% for SIS/PAA/Van@Mg, compared to 100.00±6.65% and 100.98±6.51% for SIS and SIS/PAA, respectively (Fig. 3(d, e)). These results highlighted the robust antibacterial properties of the SIS/PAA/Van@Mg bio-patch, confirming its potential for in vivo applications as an effective dural repair material.

### 3.5 Evaluating vascularization induction of the Janus adhesive bio-patch

**Fig. 4:**
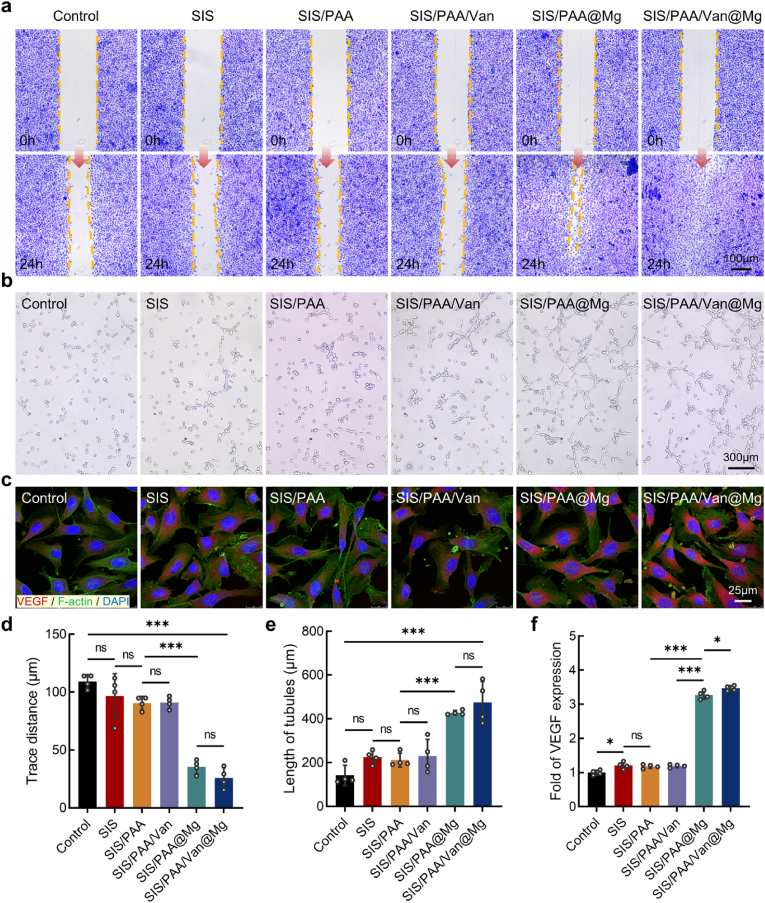
Angiogenesis study for the Janus adhesive bio-patch. (a) Cell scratch assays using HUVECs to evaluate cell migration activity. (b) Tube formation assays with HUVECs on the Matrigel, exposed to the bio-patch's extract for 24 hours. (c) Immunofluorescent staining of VEGF for seven days with the extract. Semi-quantitative analysis for (d) trace distance, (e) length of tubules, and (f) fold of VEGF fluorescent intensity based on the above panels. Statistical significance and *P* values were determined by ANOVA comparison test. The data were presented as mean ± SD (n=4). ^∗^*P*<0.05, ^∗∗∗^*P*<0.001.

Given the established role of Mg^2+^ in promoting vascularization [23, 39], we prepared the bio-patch extract following ISO10993 guidelines to evaluate its effects on HUVECs. The vascularization potential of the bio-patch was assessed through cell scratch assays, tube formation assays, and immunofluorescent staining. In the scratch assay, a uniform scratch width of 200μm was created using pipette tips. After 24 hours of co-culture with the extract, minimal cell migration was observed in the control, SIS, SIS/PAA, and SIS/PAA/Van groups, with measured trance distances of 109.11±5.90μm, 96.74±19.16μm, and 90.54±5.73μm, respectively. In contrast, the SIS/PAA@Mg and SIS/PAA/Van@Mg groups demonstrated significantly enhanced cell migration, with trace distances of 35.44±5.90μm and 25.89±8.90μm, respectively (Fig. 4(a, d)). In the tube formation assay, the SIS/PAA@Mg and SIS/PAA/Van@Mg showed substantial increase in tubular network formation compared to the other groups (Fig. 4b). The SIS/PAA/Van@Mg group exhibited the most pronounced effect, with tubular length reaching 474.84±95.54μm, which was 3.34-fold and 2.11-fold higher than the control and SIS groups, respectively (Fig. 4e). Furthermore, immunofluorescent staining for VEGF confirmed significantly higher fluorescent intensities in the SIS/PAA@Mg and SIS/PAA/Van@Mg groups, showing increase of 3.27-fold and 3.47-fold relative to the control, respectively (Fig. 4(c, f)). Interestingly, the SIS group also displayed some up-regulation of VEGF expression compared to the control, which might be attributed to residual TGF-β and FGF factors in SIS that promoted vascular differentiation [40]. These results demonstrated that the SIS/PAA/Van@Mg bio-patch possessed significantly enhanced vascularization, highlighting its potential to support tissue regeneration in dural repair applications.

### 3.6 In vivo antibacterial efficacy of the Janus adhesive bio-patch

**Fig. 5:**
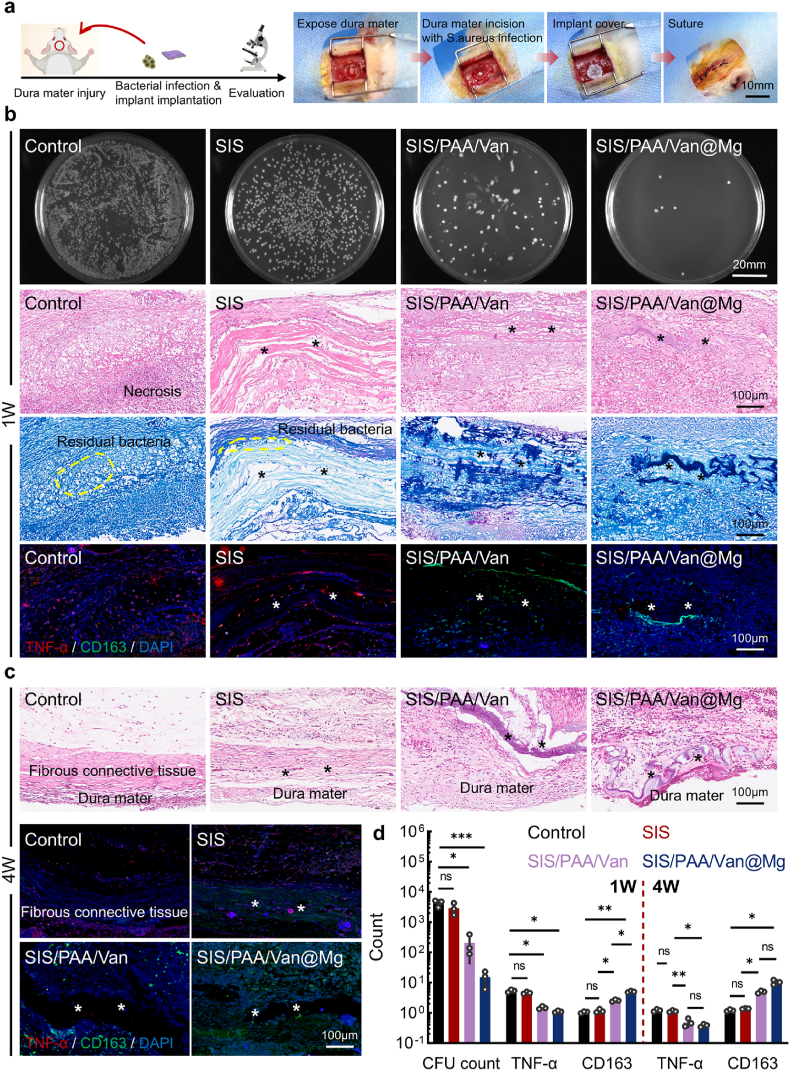
Antibacterial study for the Janus adhesive bio-patch in rat dural injury model. (a) Illustration for the animal experiment from establishing the dural injury, to introducing exogenous *S.aureus* to simulate infection and applying bio-patches to address inflammations. (b) Bacterial colony reformation, H&E staining, Giemsa staining and immunofluorescent staining of TNF-α (red), CD163 (green), DAPI (blue) after one-week surgery. (c) H&E staining, and TNF-α (red), CD163 (green), DAPI staining after four-week surgery. (d) Semi-quantitative analysis for the CFU counts, TNF-α and CD163 expression after surgery. The asterisk indicates the implanted bio-patch. Statistical significance and *P* values were determined by ANOVA comparison test. The data were presented as mean ± SD (n=3). ^∗^*P*<0.05, ^∗∗^*P*<0.01, ^∗∗∗^*P*<0.001. (For interpretation of the references to colour in this figure legend, the reader is referred to the Web version of this article.)

To further validate the in vivo antibacterial efficacy of the bio-patches, we utilized a rat dural injury model to assess their repair effectiveness in presence of exogenous *S.aureus*. In this model, rats were anesthetized, and a 5mm circular defect was carefully established in the skull. The dura mater was subsequently incised, and *S.aureus* along with the respective bio-patches were introduced into the defect site before suturing (Fig. 5a). One-week after surgery, the rats were euthanized, and granulation tissue (1mm^3^) from the injury site was harvested, soaked in sterile saline for two hours, and then plated on sterile agar for bacterial culture at 37°C for 24 hours. As shown in Fig. 5b, substantial bacterial colony formation was observed in the control and SIS groups, whereas the least bacterial growth detected in the SIS/PAA/Van@Mg group, suggesting that the synergistic release of vancomycin and Mg^2+^ effectively enhanced antibacterial activity. Histological analysis with H&E staining at one-week revealed considerable tissue necrosis in the control group, due to bacterial exotoxin release [41, 42]. Giemsa staining further confirmed the presence of residual *S.aureus* in the control and SIS groups. In contrast, no significant inflammatory response or bacterial remnants were observed in the SIS/PAA/Van and SIS/PAA/Van@Mg groups. Notably, pro-inflammatory marker TNF-α expression in the SIS/PAA/Van@Mg group was reduced to 0.21-fold to the control, while anti-inflammatory marker CD163 expression increased 4.78-fold compared to the control. At four-week after surgery, the control and SIS groups exhibited dense fibrous tissue formation, resulting from bacterial infiltration, whereas the SIS/PAA/Van and SIS/PAA/Van@Mg groups displayed normal cellular infiltration into the bio-patch. Notably, CD163 expression in the SIS/PAA/Van@Mg group was 8.60-fold higher than the control and 7.54-fold higher than the SIS group (Fig. 5(c, d)). These results demonstrated the superior in vivo antibacterial efficacy and biocompatibility of the SIS/PAA/Van@Mg bio-patch, underscoring its potential as a promising solution for effective dural repair.

### 3.7 Dural repair by the Janus adhesive bio-patch in rabbits

**Fig. 6:**
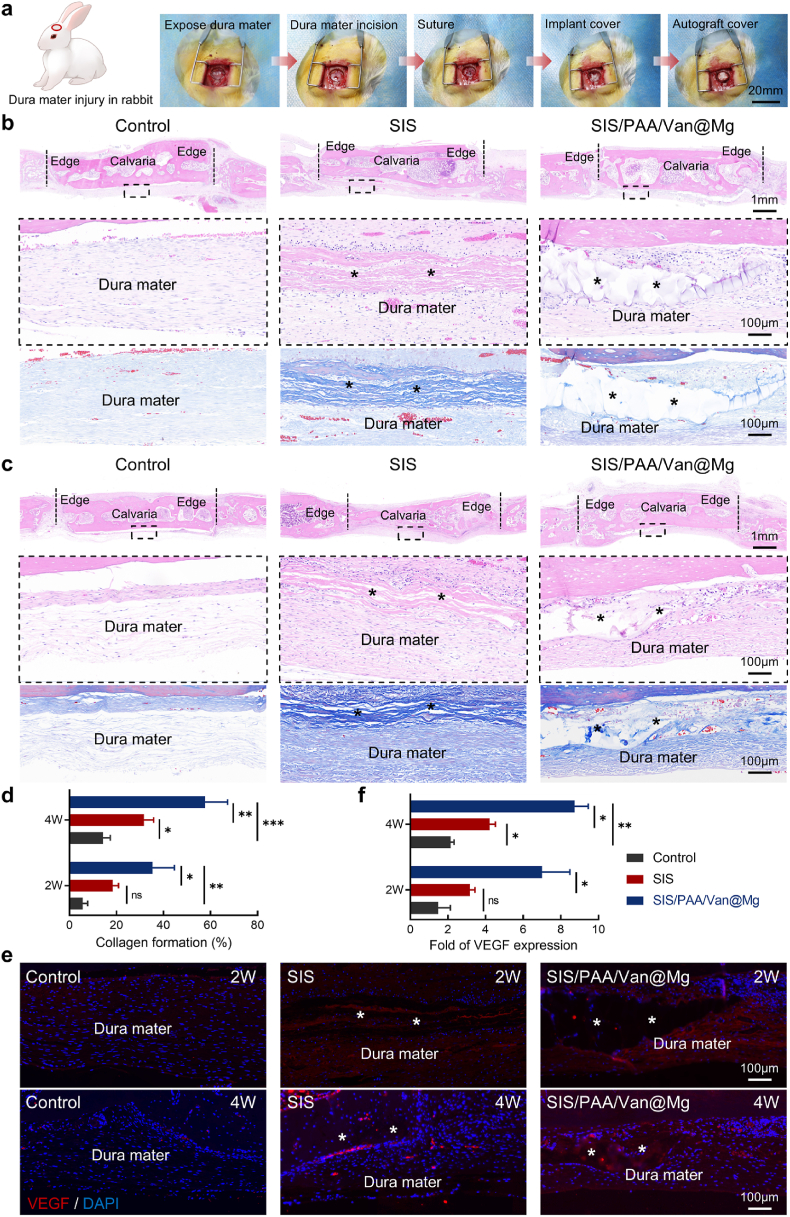
Dural repair for the Janus adhesive bio-patch in rabbit model. (a) Illustration for the animal experiment from establishing the dural injury, to implanting the bio-patch to promote the wound regeneration. H&E and Masson's trichrome staining at (b) two-week, (c) four-week after surgery. (d) Estimate collagen formation at the located sites. (e) Immunofluorescent staining of VEGF (red) in the rabbit models after two- and four-week after surgery. (f) Quantitative analysis for VEGF expressions. The asterisk indicates the implanted bio-patch. Statistical significance and *P* values were determined by ANOVA comparison test. The data were presented as mean ± SD (n=3). ^∗^*P*<0.05, ^∗∗^*P*<0.01, ^∗∗∗^*P*<0.001. (For interpretation of the references to colour in this figure legend, the reader is referred to the Web version of this article.)

To evaluate the tissue repair capabilities of the Janus adhesive bio-patch, we employed a New Zealand rabbit model with a 10mm skull defect created during craniotomy to simulate dural mater injury. The defect was initially closed with sutures (2–3 stitches), followed by implantation of the SIS-based dural repair bio-patch. Autologous bone grafts were placed over the defect, and the wound was subsequently sutured (Fig. 6a). Histological evaluations at two-week after surgery, using H&E and Masson's trichrome staining revealed no obvious inflammation in either the SIS and SIS/PAA/Van@Mg implant groups (Fig. 6b). At four-week after surgery, the implanted bio-patches exhibited gradual degradation, with surrounding dura mater tissue displaying robust collagen deposition (Fig. 6c). Quantitative analysis showed that collagen deposition in the SIS/PAA/Van@Mg group reached 35.33±9.29 % at two-week, and 57.67±9.61 % at four-week. In comparison, the control and SIS groups demonstrated significantly lower collagen formation, with values of 14.33±3.06 % and 31.67±4.16 % at four-week, respectively (Fig. 6d). VEGF fluorescence staining displayed a remarkable increase in vascularization induced by the SIS/PAA/Van@Mg implants at two-week, with VEGF expression levels reaching 6.08-fold higher than the control. This elevated VEGF expression remained significantly higher at four-week, persisting at a 4.08-fold compared to the control.

To further assess tissue repair progression, we analyzed inflammatory and repair-related markers, including TNF-α and TGF-β. At two-week post-implantation, the SIS/PAA/Van@Mg group exhibited significantly reduced TNF-α levels (0.16-fold lower than the control), indicating diminished inflammation. Concurrently, TGF-β expression in the SIS/PAA/Van@Mg group was elevated to 3.64-fold higher than the control. At four-week after implantation, TGF-β levels in the SIS/PAA/Van@Mg group remained elevated, at 2.31-fold higher than the control and 1.38-fold higher than the SIS group (Fig. S3).

To further evaluate the biocompatibility and safety of the bio-patches, we conducted a comprehensive examination of major organs including heart, liver, spleen, lung, and kidney, as well as hematological assessments at four-week after implantation. Histological analysis showed no notable adverse effects in the SIS/PAA/Van@Mg group, with no abnormality observed. Hematological parameters, such as white blood cell (WBC), red blood cell (RBC), alanine aminotransferase (ALT), and aspartate aminotransferase (AST) were also measured. The results indicated no significant difference between the SIS/PAA/Van@Mg and the control (Fig. S4, Fig. S5), suggesting that the SIS/PAA/Van@Mg implants did not trigger any systemic immune or toxic reactions. These findings confirmed that the bio-patch did not elicit any systemic immune response or toxic reaction. These findings confirmed the excellent biocompatibility and safety of the SIS/PAA/Van@Mg bio-patch, demonstrating no detrimental effect on vital organ function or hematological parameters. Combined with their proven efficacy in promoting tissue regeneration, the SIS/PAA/Van@Mg bio-patches held significant promise for safe and efficient dural repair applications.

### 3.8 Pre-clinical evaluating the Janus adhesive bio-patch in beagle dogs

**Fig. 7:**
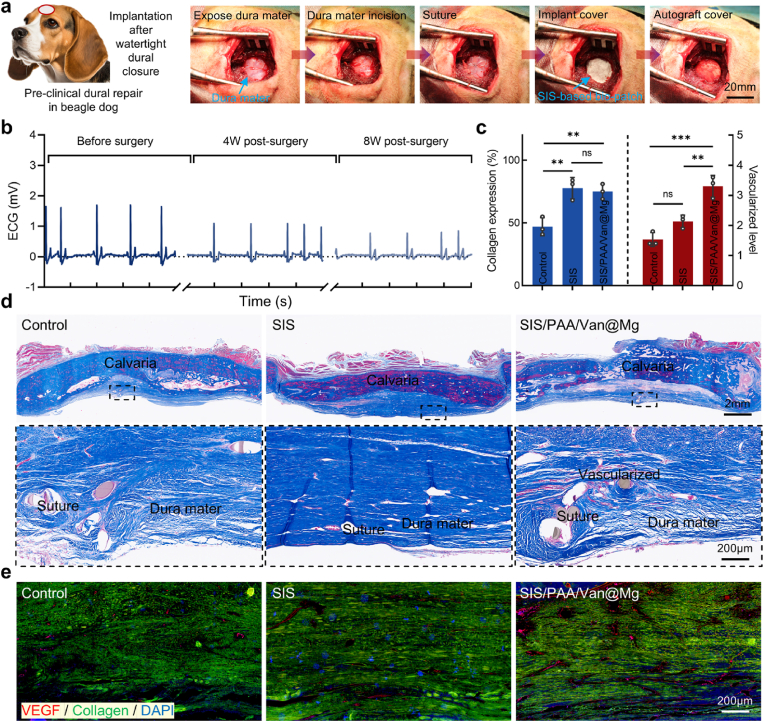
Pre-clinical trials of dural repair for the Janus adhesive bio-patch using beagle dog. (a) Illustration for the animal experiment from establishing the dural injury, to implanting the bio-patch to promote dura mater regeneration. (b) ECG curves along with the SIS/PAA/Van@Mg bio-patch implanted at different intervals. (c) Collagen expression, and vascularized level at eight-week surgery. (e) Immunofluorescent of VEGF (red), collagen (green), DAPI (blue) at the located wound sites at eight-week post-surgery. Statistical significance and *P* values were determined by ANOVA comparison test. The data were presented as mean ± SD (n=3). ^∗∗^*P*<0.01, ^∗∗∗^*P*<0.001. (For interpretation of the references to colour in this figure legend, the reader is referred to the Web version of this article.)

To comprehensively evaluate the clinical efficacy and safety of the SIS/PAA/Van@Mg bio-patch, we consulted medical device registration specialists and clinical practitioners. Based on their input, we modified our study to align more closely with clinical procedure and conducted a detailed investigation using Beagle dogs. This study followed a methodology similar to our experiments on New Zealand rabbits, with adjustments to better replicate human clinical practices. In this experiment, a cranial defect was created in the Beagle dogs, followed by a ∼2cm sagittal incision at the dura mater's base. Standard clinical procedures were then employed to achieve a watertight primary closure of the dural mater using sutures [43]. The SIS/PAA/Van@Mg bio-patch was subsequently applied for secondary sealing, followed by reimplantation of autologous bone, and the wound was closed with layered sutures (Fig. 7a). To assess cardiac safety, ECG monitoring was performed at pre-surgery, four-week, and eight-week after surgery. Initial results showed normal cardiac rhythms, with regular R waves and heart rates within the physiological range. While minor transient alterations in cardiac electrical activity were observed at four-week post-surgery, cardiac function returned to baseline by eight-week, indicating good cardiac safety (Fig. 7b). At eight-week after implantation, the animals were euthanized, and histological analysis of the dura mater repair sites was performed. Masson's trichrome staining and VEGF/collagen/DAPI immunofluorescence staining revealed the denser and more organized collagen fibers in the SIS/PAA/Van@Mg group, with well-aligned collagen and abundant blood vessels in the regenerated dura mater. Quantitative analysis demonstrated that the collagen content in the SIS/PAA/Van@Mg group was 75.00±6.00%, significantly higher than the control group (47.00±7.21%). Furthermore, vascularization levels in the SIS/PAA/Van@Mg groups were 2.15-fold higher than the control (Fig. 7(c-e)). These results strongly supported the potential clinical utility of the SIS/PAA/Van@Mg bio-patch as an effective material for dural repair, demonstrating both safety and enhanced efficacy in promoting tissue regeneration and vascularization.

Additionally, at eight-week post-surgery, the SIS/PAA/Van@Mg bio-patch exhibited excellent biocompatibility and biosafety. Histological evaluations of the implantation sites revealed no significant inflammatory or immune rejection (Fig. S6, Fig. S7). Pathological analysis of major organs, including the heart, liver, spleen, lung, and kidney, showed no abnormality, further confirming the absence of systemic adverse effects. These findings reinforced the SIS/PAA/Van@Mg bio-patch's suitability for safety and effective clinical application in dural repair, positioning it as a promising candidate for future neurosurgical use.

## 4 Conclusions

In this study, we developed a Janus adhesive bio-patch with targeted drug delivery capabilities for dura mater repair. By incorporating vancomycin and MgCO_3_ into a PAA hydrogel coating on a SIS extracellular matrix substrate, we constructed a dual-functional material with robust antibacterial (antibacterial efficiency ∼99.17%) and pro-angiogenic (3.47-fold higher than the control) properties. The bio-patch exhibited sufficient burst strength (∼20.50kPa) to effectively seal dura mater injuries and prevent CSF leakage. In vivo animal studies conducted in rat, rabbit, and beagle dog dura mater injury models further demonstrated the bio-patch's significant therapeutic potential, including enhancing tissue regeneration, effective CSF leak prevention, and superior biocompatibility. These findings underscored the SIS/PAA/Van@Mg bio-patch as a promising, versatile, and effective solution for improving clinical outcomes in neurosurgical applications, offering a new strategy for safe and efficient dura mater repair.

## CRediT authorship contribution statement

**Yirizhati Aili:** Writing – original draft, Investigation, Conceptualization. **Pengfei Wei:** Investigation, Data curation. **Xueqiao Yu:** Investigation, Conceptualization. **Guofeng Fan:** Formal analysis. **Nuerailijiang Maimaitiaili:** Investigation. **Yunhuan Li:** Investigation. **Siqi Liu:** Formal analysis. **Yiqian Huang:** Validation. **Bo Zhao:** Supervision. **Zengliang Wang:** Validation, Methodology. **Hu Qin:** Supervision. **Yongxin Wang:** Writing – review & editing, Supervision, Project administration.

## Declaration of competing interest

The authors declare that they have no known competing financial interests or personal relationships that could have appeared to influence the work reported in this paper.

## Data Availability

Data will be made available on request.
