# Supplementary material for: Janus adhesive bio-patches with targeted drug delivery enabled anti-bacteria and pro-angiogenesis for dura mater repair
Source: Mater Today Bio. 2025 Jan 11;31:101484. doi: 10.1016/j.mtbio.2025.101484 (PMC11804716; doi:10.1016/j.mtbio.2025.101484)
Supplement: Multimedia component 1 [file mmc1.docx]

**Supplementary Materials**

**Janus adhesive bio-patches with targeted drug delivery enabled anti-bacteria and pro-angiogenesis for dura mater repair**

Yirizhati Aili^1,2,#^, Pengfei Wei^3,#^, Xueqiao Yu^3,#^, Guofeng Fan^1^, Nuerailijiang Maimaitiaili^1^, Yunhuan Li^3^, Siqi Liu^3^, Yiqian Huang^3^, Bo Zhao^3,*^, Zengliang Wang^1,2,*^, Hu Qin^1,*^, Yongxin Wang^1,2,*^

^1^ Department of Neurosurgery, The First Affiliated Hospital of Xinjiang Medical University, No.393 Xinyi Road, Urumqi, Xinjiang, 830054, China

^2^ Key Laboratory of Precision Diagnosis and Clinical Transformation of Nervous System Tumors, Xinjiang Medical University, No.393 Xinyi Road, Urumqi, Xinjiang, 830054, China

^3^ Beijing Biosis Healing Biological Technology Co., Ltd. No.29 Yongda Road, Beijing, 102600, China

^#^ These authors contribute equally to this work.

^*^ Corresponding authors.

E-mail: zhaobo@biosishealing.com (B. Zhao), wzl3ng@126.com (Z. Wang), qinhu86@163.com (H. Qin), xjdwyx2000@sohu.com (Y. Wang).


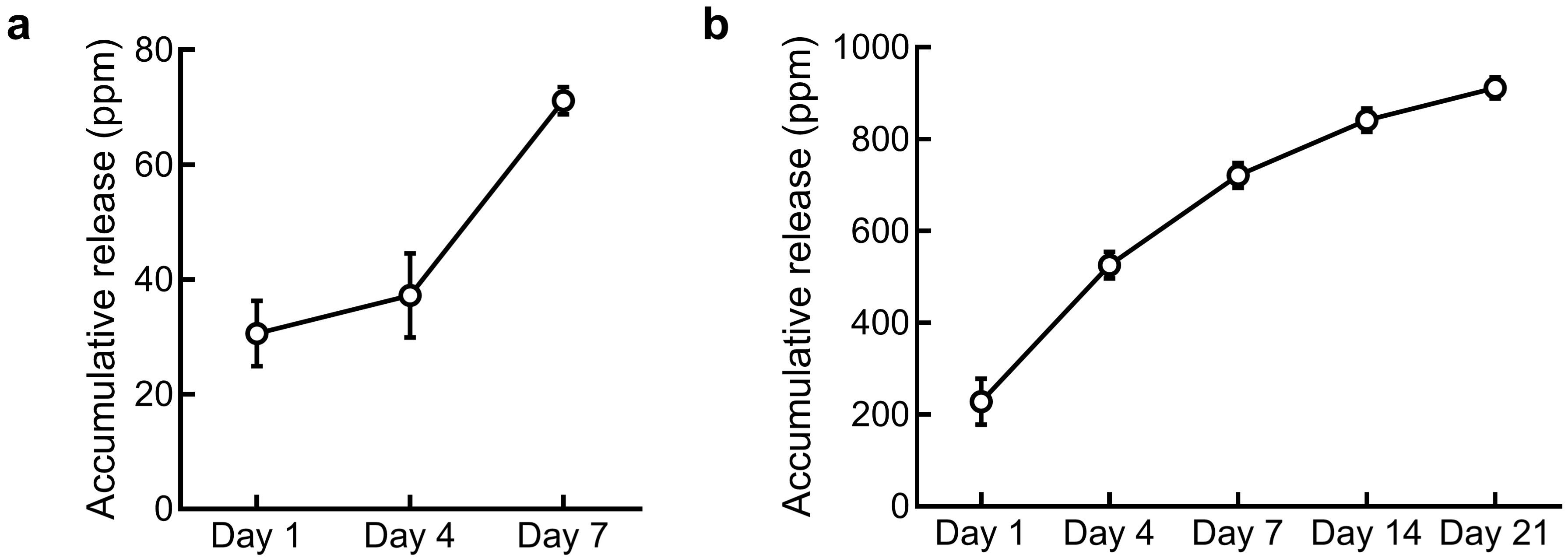


**Fig. S1.** Accumulative release of (a) vancomycin, and (b) Mg^2+^ at from the SIS/PAA/Van@Mg bio-patch at 37℃.


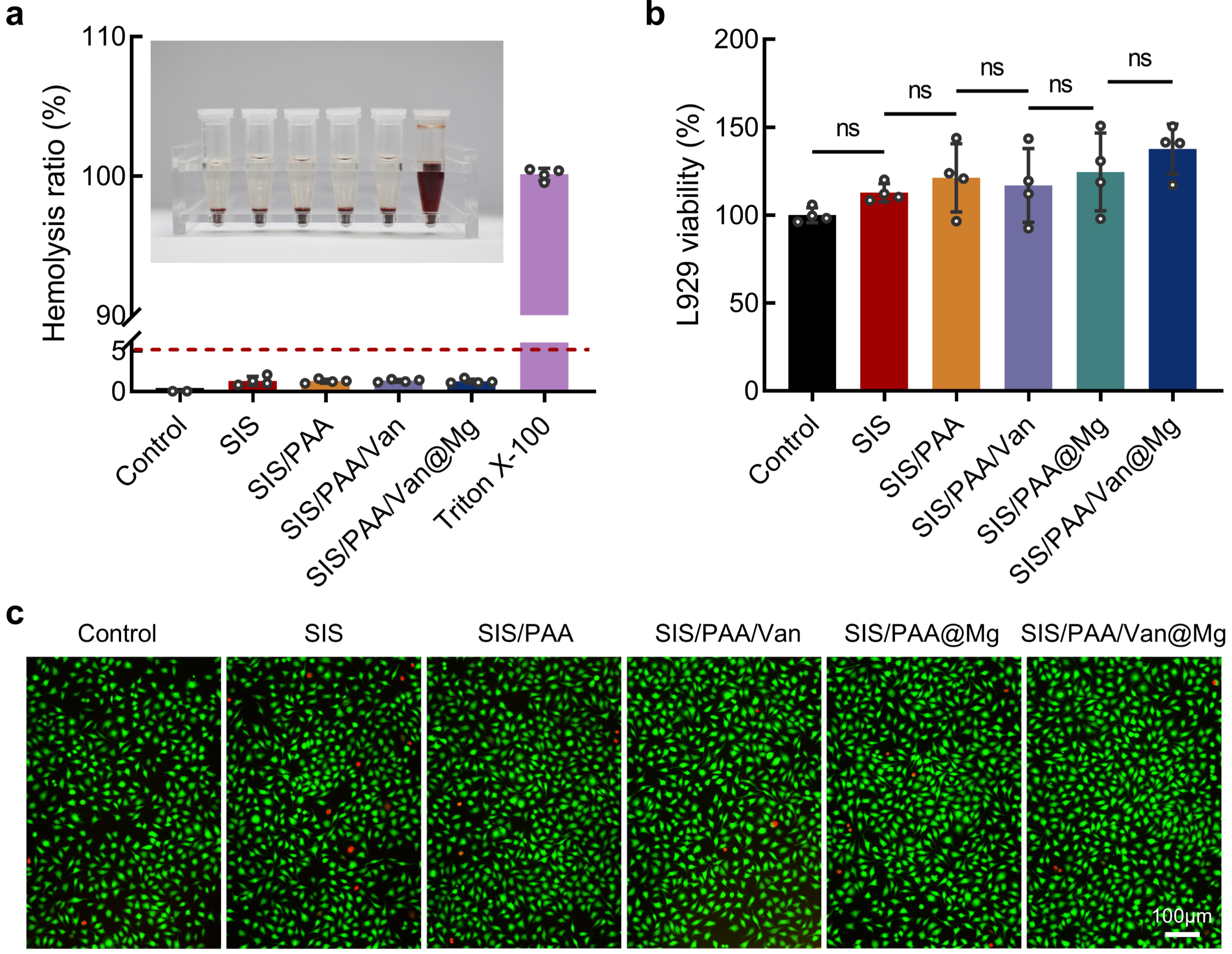


**Fig. S2.** Biocompatibility study for the Janus adhesive bio-patch. (a) Hemolysis study with red cells (inset image depicts the appearance of the supernatants). (b) Quantitative analysis of L929 fibroblast viability when growing with the bio-patch’s extract for 24 hours. (c) Live/dead staining using calcein-AM/PI with L929 for 24-hour growth. Statistical significance and *P* values were determined by ANOVA comparison test. The data were presented as mean ± SD (n=4).


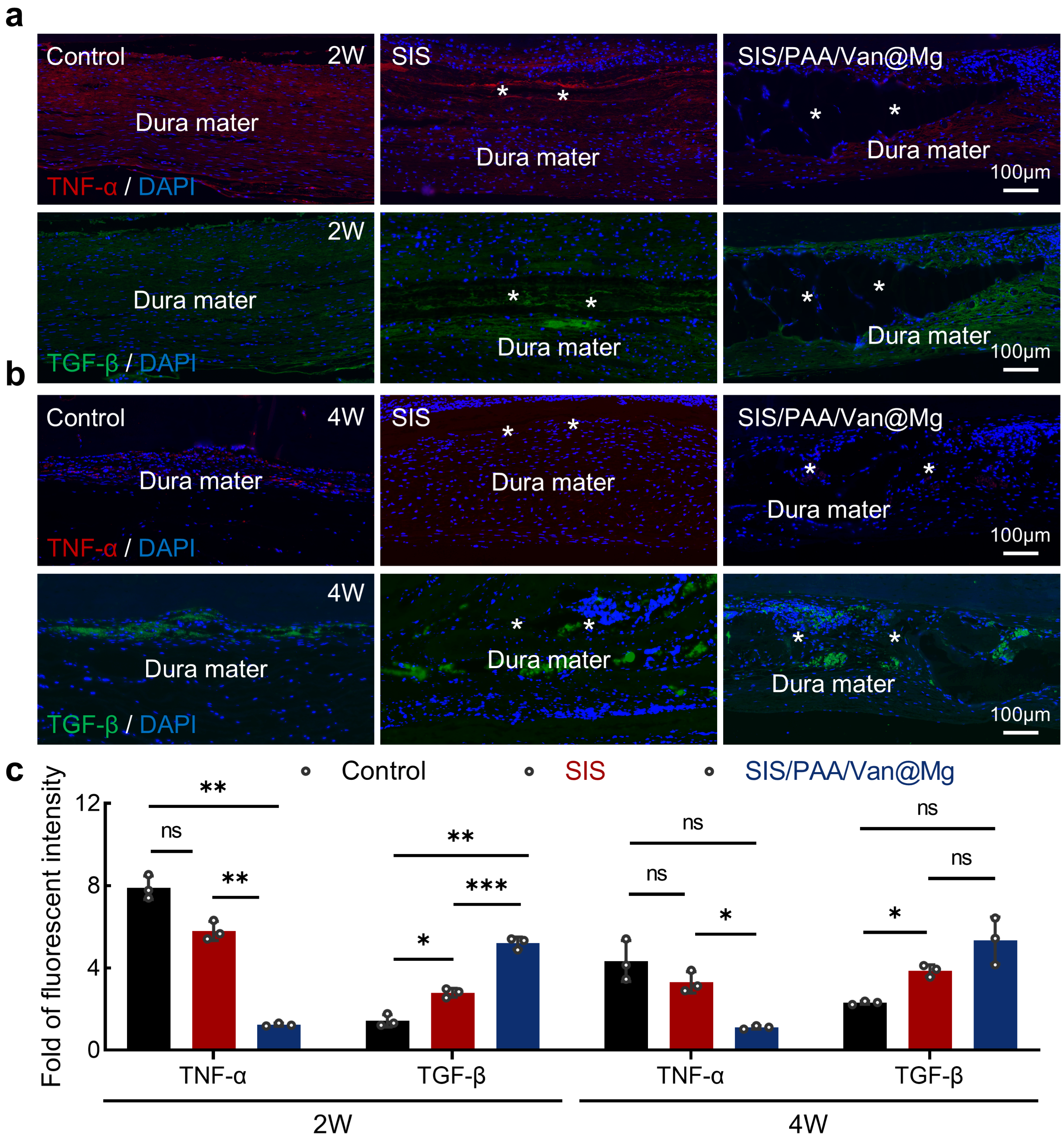


**Fig. S3.** Immunofluorescent staining of TNF-α (red), TGF-β (green) and VEGF (red) in the rabbit models after (a) two-week surgery, and (b) four-week surgery. (c) Quantitative analysis for the TNF-α, TGF-β and VEGF expressions. The asterisk indicates the implanted bio-patch. Statistical significance and *P* values were determined by ANOVA comparison test. Statistical significance and *P* values were determined by ANOVA comparison test. The data were presented as mean ± SD (n=3). ^*^*P*≤0.05, ^**^*P*≤0.01, ^***^*P*≤0.001.


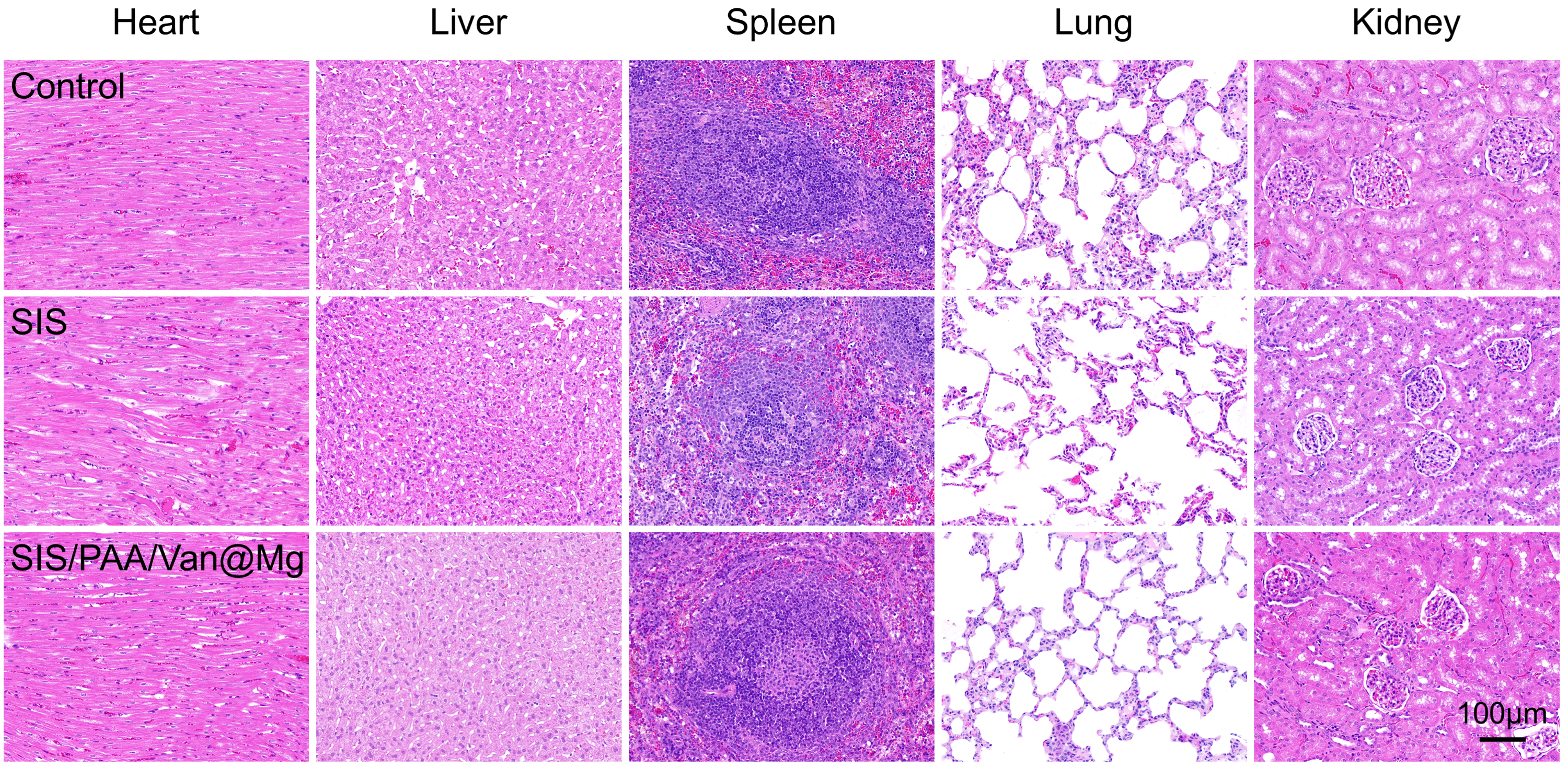


**Fig. S4.** H&E staining for the heart, liver, spleen, lung and kidney in the rabbit models after four-week post-surgery.


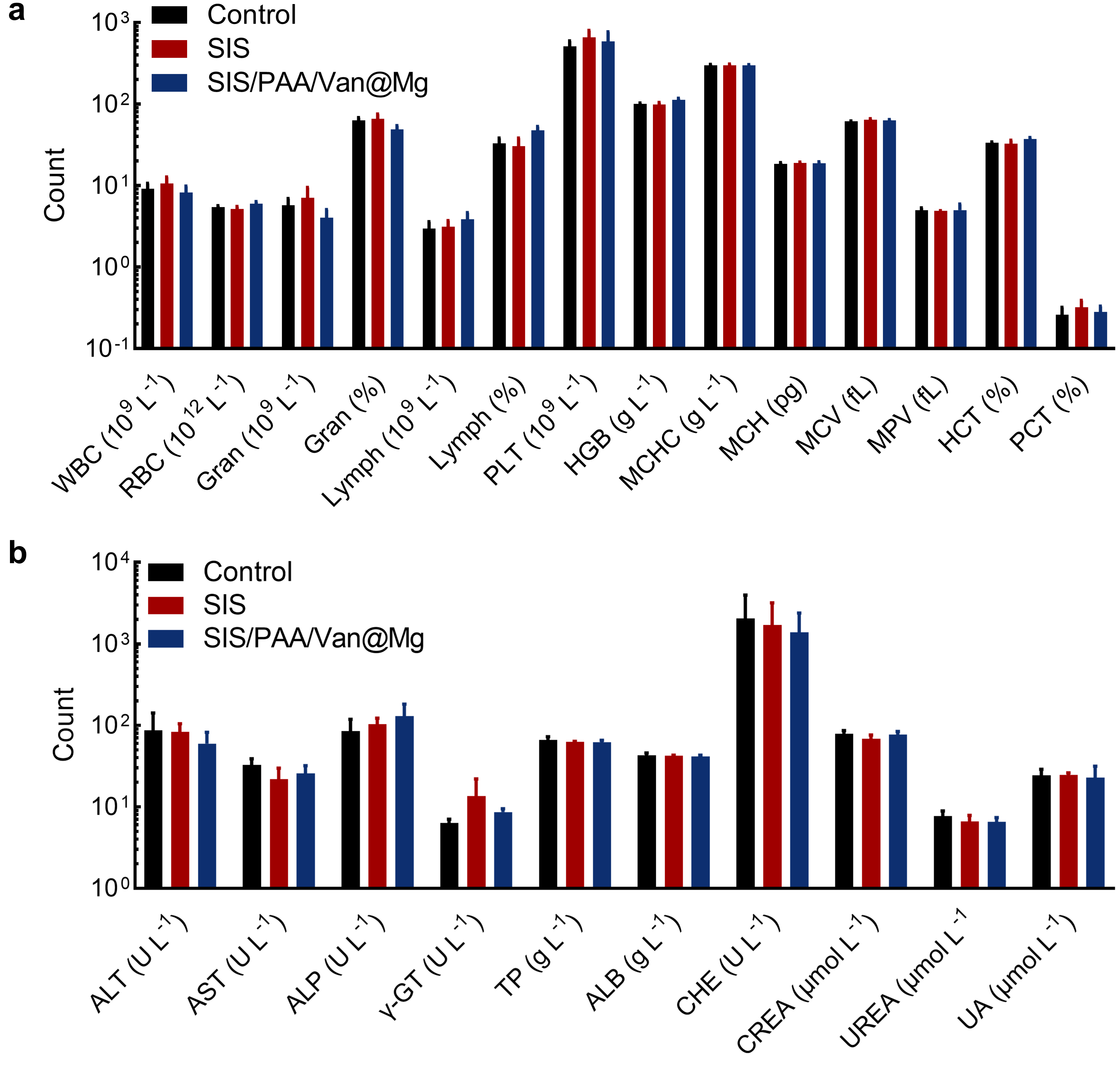


**Fig. S5.** Hematological study for (a) complete blood cell count, and (b) blood chemistry in the rabbit models after four-week surgery.


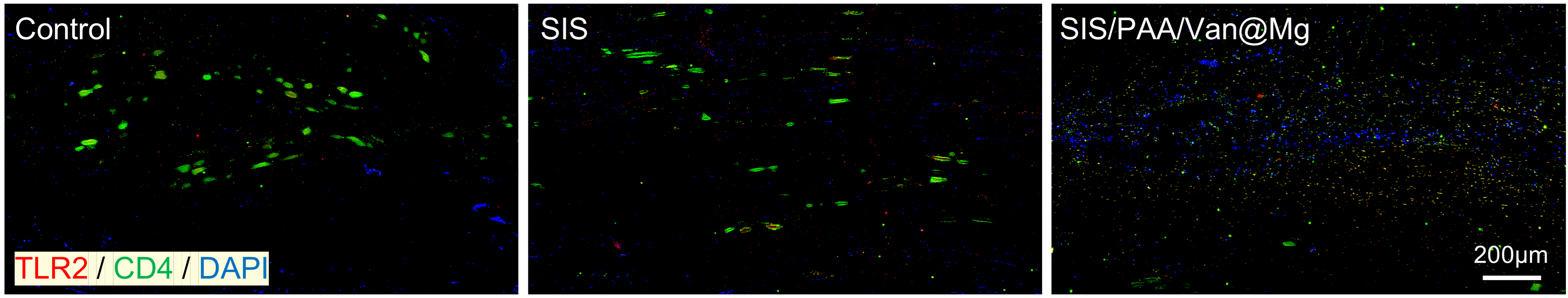


**Fig. S6.** Immunofluorescent of TLR2 (red), CD4 (green), and DAPI (blue) at the located wound sites at eight-week surgery.


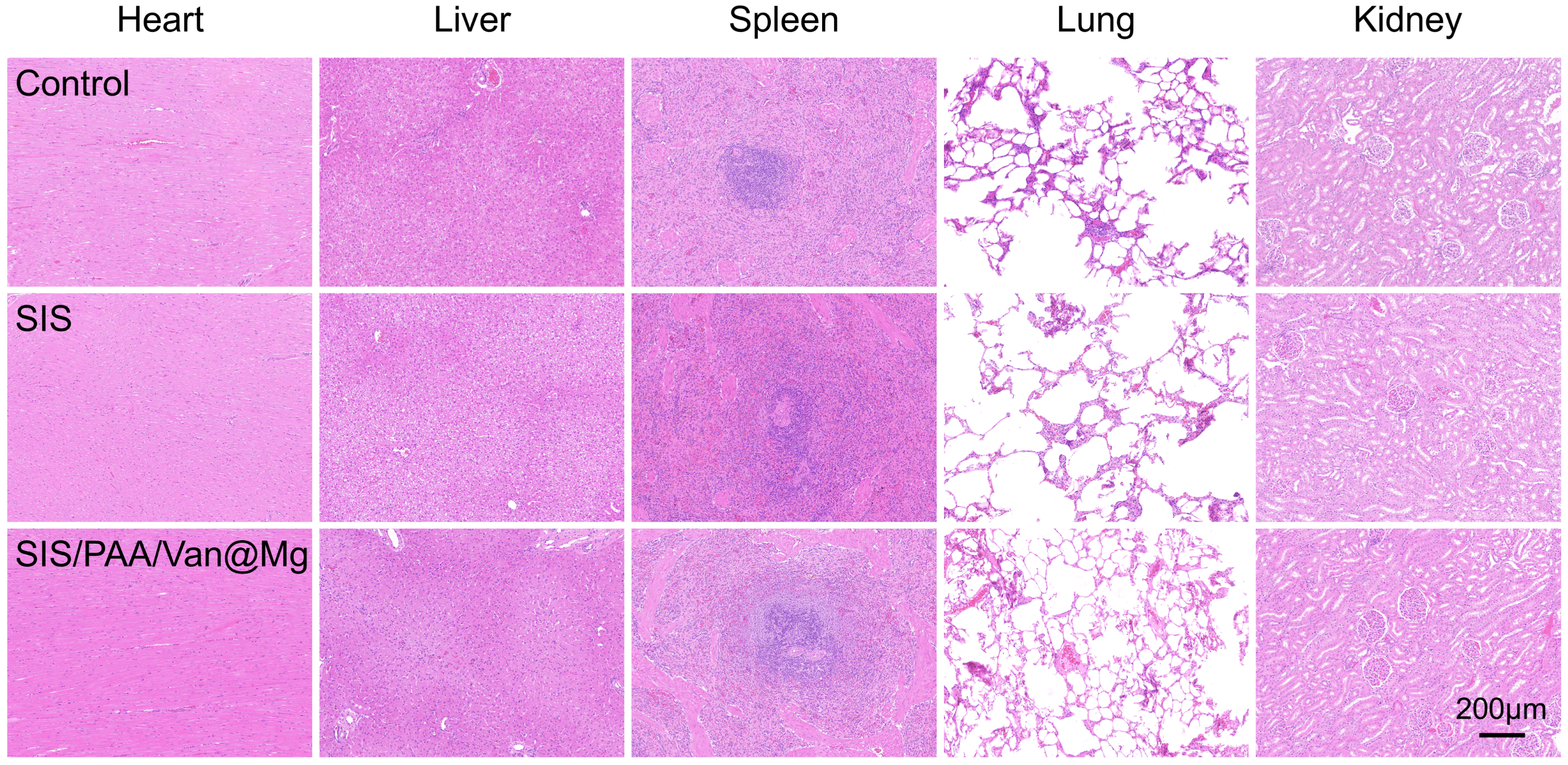


**Fig. S7.** H&E staining for the heart, liver, spleen, lung and kidney in the beagle models after eight-week surgery.
